# Development of the ASSESS tool: a comprehenSive tool to Support rEporting and critical appraiSal of qualitative, quantitative, and mixed methods implementation reSearch outcomes

**DOI:** 10.1186/s43058-021-00236-4

**Published:** 2022-03-28

**Authors:** Nessa Ryan, Dorice Vieira, Joyce Gyamfi, Temitope Ojo, Donna Shelley, Olugbenga Ogedegbe, Juliet Iwelunmor, Emmanuel Peprah

**Affiliations:** 1 Global Health Program, New York University School of Global Public Health, Public Health, 708 Broadway, 4th floor - Room 453, New York, NY 10003 USA; 2 NYU Health Sciences Library, Grossman School of Medicine, New York University, New York, NY USA; 3 Department of Social and Behavioral Sciences, New York University School of Global Public Health, New York, NY USA; 4 Department of Public Health Policy and Management, New York University School of Global Public Health, New York, NY USA; 5 Department of Population Health, NYU School of Medicine, NYU Langone Health, New York, NY USA; 6 Behavioral Science and Health Education, College for Public Health and Social Justice, Salus Center, Saint Louis University, Saint Louis, MO USA

**Keywords:** Reporting tool, Critical appraisal, Implementation outcomes, Implementation strategies, Qualitative methods, Quantitative methods, Mixed methods, Systematic review, Meta-analysis

## Abstract

**Background:**

Several tools to improve reporting of implementation studies for evidence-based decision making have been created; however, no tool for critical appraisal of implementation outcomes exists. Researchers, practitioners, and policy makers lack tools to support the concurrent synthesis and critical assessment of outcomes for implementation research. Our objectives were to develop a comprehensive tool to (1) describe studies focused on implementation that use qualitative, quantitative, and/or mixed methodologies and (2) assess risk of bias of implementation outcomes.

**Methods:**

A hybrid consensus-building approach combining Delphi Group and Nominal Group techniques (NGT) was modeled after comparative methodologies for developing health research reporting guidelines and critical appraisal tools. First, an online modified NGT occurred among a small expert panel (*n* = 5), consisting of literature review, item generation, round robin with clarification, application of the tool to various study types, voting, and discussion. This was followed by a larger e-consensus meeting and modified Delphi process with implementers and implementation scientists (*n* = 32). New elements and elements of various existing tools, frameworks, and taxonomies were combined to produce the ASSESS tool.

**Results:**

The 24-item tool is applicable to a broad range of study designs employed in implementation science, including qualitative studies, randomized-control trials, non-randomized quantitative studies, and mixed methods studies. Two key features are a section for assessing bias of the implementation outcomes and sections for describing the implementation strategy and intervention implemented. An accompanying explanation and elaboration document that identifies and describes each of the items, explains the rationale, and provides examples of reporting and appraising practice, as well as templates to allow synthesis of extracted data across studies and an instructional video, has been prepared.

**Conclusions:**

The comprehensive, adaptable tool to support both reporting and critical appraisal of implementation science studies including quantitative, qualitative, and mixed methods assessment of intervention and implementation outcomes has been developed. This tool can be applied to a methodologically diverse and growing body of implementation science literature to support reviews or meta-analyses that inform evidence-based decision-making regarding processes and strategies for implementation.

Contributions to the literature

- The ASSESS tool addresses the challenge of critical assessment of a methodologically diverse and growing body of implementation science literature.
- This tool is helpful for designing and executing reviews and meta-analyses of empirical studies of implementation, examining how process and context may lead to heterogeneity of results.
- Its use standardizes the reporting and synthesis of implementation strategies, which will facilitate translation of effective public health interventions into routine practice within clinical or community settings.

## Background

Implementation research applies a diverse range of study designs to increase translation of research evidence into policies and practice [1–7]. It allows us to conceptualize and evaluate successful implementation of interventions, particularly via assessment of implementation outcomes, which are the effects of implementation strategies, or deliberate and purposive actions to implement a new treatment, practice, or service [8]. As a poorly implemented program or policy will not have the intended interventional impact [8], robust implementation outcomes are also crucial to achieve the desired population health impact [8–10]. Implementation science studies may use quantitative, qualitative, and/or mixed-methodologies to assess these implementation outcomes (i.e., acceptability, adoption, appropriateness, cost, feasibility, fidelity, penetration, or sustainability) or intervention outcomes (i.e., effectiveness, efficiency, equity, patient-centeredness, safety, or timeliness), particularly within hybrid effectiveness-implementation designs [1]. However, researchers, practitioners, and policy makers lack tools to support the concurrent synthesis and critical assessment of implementation outcomes. Tools are needed that can support systematic reviews or meta-analyses comparing multiple types of implementation outcomes across diverse study designs.

No tool to support critical assessment of implementation outcomes exists. The product of the process of critical assessment is knowledge, usually based on appraisal of study methods that provides a level of confidence in study findings. This is an important part of evidence-based decision making, as having only an understanding of the magnitude of the success of an intervention and its implementation without an understanding of one’s confidence in study findings limits the capacity for knowledge translation. Ultimately, comprehensive identification, synthesis, and appraisal of implementation outcomes, will improve understanding of implementation processes and allow comparison of the effectiveness of different implementation strategies. Indeed, previous research has shown the need for pragmatic measures in implementation practice (including assessment of implementation context, processes, and outcomes) [11] that should be useful, compatible, acceptable, and easy [12]. Researchers have established there remains a dearth of psychometrically valid survey assessment tools for implementation outcomes, and this area of investigation is ongoing [13, 14]. Some efforts have been made to generate valid, brief assessment surveys for feasibility, acceptability, and appropriateness [15].

A tool is needed to support systematic reviews and meta-analyses of studies using qualitative, quantitative, and/or mixed methods assessment to inform evidence-based decision making on implementation. We have developed ASSESS, a comprehensive 24-item tool that (1) can describe studies evaluating implementation outcomes using qualitative, quantitative, and/or mixed methodologies and (2) can provide a rubric to grade the risk of bias of implementation outcomes.

## Methods

The development of the ASSESS tool was modeled after recommended methodologies for developing health research reporting guidelines and critical appraisal tools [16–19]. A completed checklist of the recommended steps for developing a health research reporting guideline is available as an additional file [16]. We utilized a hybrid consensus-building approach combining e-Delphi Group and Nominal Group techniques (NGT). This approach builds on the strengths of these different techniques, namely the opportunity for discussion and efficient information exchange among a smaller group of experts that is characteristic of the NGT and the process of structured documentation and consensus meeting with a larger group that is characteristic of the Delphi method [18, 19].

**Fig. 1:**
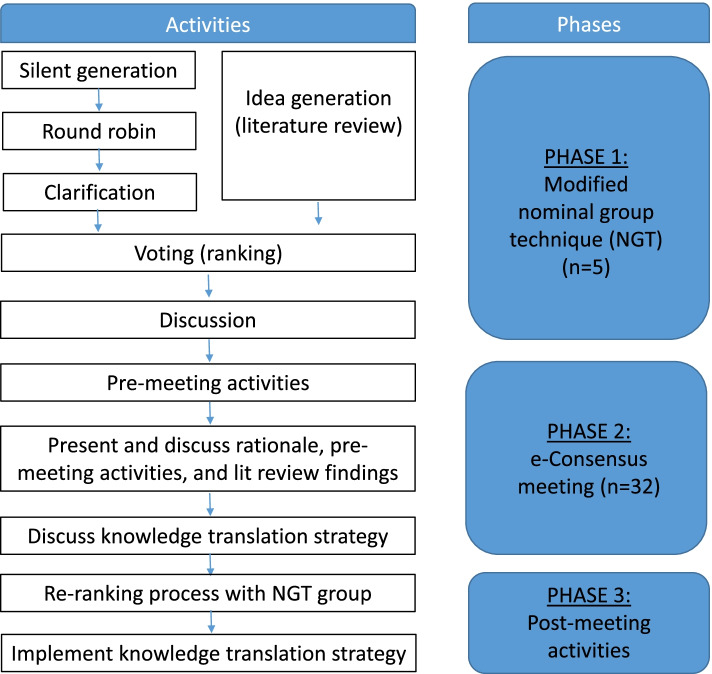
Phases of modified nominal group technique and e-consensus meeting. Adapted from McMillan SS, King M, Tully MP. How to use the nominal group and Delphi techniques. Int J Clin Pharm. 2016;38(3):655-662. doi:10.1007/s11096-016-0257-x and Moher D, Schulz KF, Simera I, Altman DG. Guidance for Developers of Health Research Reporting Guidelines. PLOS Medicine. 2010;7(2):e1000217

This hybrid process is mapped by phase in Fig. 1, with each phase described in detail below: an online modified NGT among a small expert panel (phase 1), an e-Consensus meeting among a larger panel (phase 2), and post-meeting activities (phase 3).

### Phase 1: Nominal group technique modified for online interaction

From February to October 2020 when social distancing guidelines for the COVID-19 pandemic prohibited in-person meetings, a panel of five public health professionals and implementation researchers carried out bimonthly online meetings using a NGT to conceptualize, reflect upon, develop, discuss, and refine the tool. The group’s experience and expertise was in epidemiology (*n* = 4); implementation science (*n* = 4); quantitative (*n* = 5), qualitative (*n* = 3), and mixed methodology (*n* = 3); and library science (*n* = 1). Different public health specialty areas included non-communicable disease, epigenetics, maternal health, and global health. Members were predominantly female (*n* = 4) and were working as faculty (*n* = 2), post-doctoral fellows (*n* = 2), or a public health doctoral candidate (*n* = 1). Phase 1 entailed reviewing the literature and brainstorming to generate items, followed by multiple rounds of independent assessment of items through structured data collection among this panel. Independent ratings were compiled, summarized, distributed, and discussed. This process continued until convergence of ratings was achieved.

#### Literature review and idea generation

Panel members conducted a thorough literature search of several databases (i.e., PubMed, PsycInfo, CINAHL, EMBASE, Web of Science, and Google Scholar) to inform the rationale for and conceptualization of the ASSESS tool. This review was carried out in February 2020 to begin the NGT process and was revisited eight months later to ensure no recent, relevant publications had been missed. Review findings included material on the development of tools for the purpose of reporting interventions [20, 21], reporting implementation strategies [20], the adaptation of interventions and/or their delivery [22], and identifying potential sources of bias in relevant studies using quantitative, qualitative, or mixed method assessment [23]. A search of the EQUATOR (Enhancing the QUAlity and Transparency Of health Research) Network’s library for health research reporting (http://www.equator-network.org) confirmed there were no tools for critical appraisal of implementation outcomes, thus solidifying the need for this tool. To develop our tool, a list of new items and items from existing tools were combined, including elements from the TIDieR checklist [21], StaRI checklist [20], MMAT tool [23], implementation outcomes taxonomy [8], and FRAME framework [22]. These tools are described briefly in Table 1. Novel elements of the tool included a section for critical appraisal of implementation outcomes and a space to indicate implementation phase (i.e., whether assessment was carried out pre-, during, or post implementation).

**Table 1:** Summary of tools integrated into the ASSESS tool

Tool	Function
TiDier checklist (Hoffman et al., 2014)	Reporting intervention studies
Stari checklist (Pinnock et al., 2017)	Reporting implementation studies
FRAME framework (Stirman et al., 2019)	Reporting adaptation to an intervention content and/or its delivery
MMAT tool (Hong et al., 2018)	Critical appraisal for mixed method studies
Taxonomy for implementation outcomes (Proctor et al., 2011)	Description of implementation outcomes

#### Round robin and clarification

After integrating existing reporting and appraisal tools with novel elements, we developed an initial shared draft of the tool in Excel 2016. During multiple online meetings, panel members were provided the opportunity to provide structured feedback on each item, its content and presentation, as well as the overall structure of the tool and its instructions. All panel members were encouraged to provide clarification on their feedback, including rationale for rankings, while one panel member took notes on a shared document (this replaced the white board upon which notes would be taken if this were an in-person meeting).

#### Voting and discussion

All panel members voted on the items to be included within the ASSESS tool and their presentation. The panel suggested four domains capturing implementation methods, intervention methods, implementation results, and intervention results. These domains include (i) intervention and implementation description: methods, (ii) intervention and implementation description: results, (iii) intervention and implementation evaluation: methods, and (iv) intervention and implementation evaluation: results. Panel members discussed the rationale for these domains: that they would allow users to fully describe the methods and results of the study relevant to the intervention and implementation strategy, as well as to critically appraise the outcomes relevant to the implementation strategy and the intervention being implemented. The team deliberated and agreed upon content, structure, and the addition of instructions and further explanation on using the tool.

Once an initial version was developed, the tool was applied by each panel member to articles representing various study types (i.e., randomized-control trials, non-randomized quantitative studies, qualitative studies, and mixed methods studies) and studies representing various phases of implementation. In between meetings, all panel members would apply the tool to the same articles as other panel members and take notes on that experience. Then, during meetings, the NGT process would be repeated with periods of generation of suggested modifications, round robin, clarification, voting, and discussion. Modifications made to enhance the tool as needed based on results from this process included adding further explanation of items and re-ordering the presentation of items for clarity.

#### Additional expert feedback

Additional expert feedback was invited on draft versions of the ASSESS tool. A draft version of the tool was shared via e-mail, along with a suggested article for application of the tool, with two experts for feedback. These experts reviewed and provided significant feedback before seeking further feedback via a larger group e-Consensus meeting. Experts shared suggestions for adding further explanation on the critical appraisal section and for re-formatting instructions for clarity.

### Phase 2: e-Consensus meeting

After the iterative process incorporating feedback from panel experts and additional experts, we sought feedback from hypothetical users of the tool. Implementation researchers and implementers (*N* = 32) were recruited via email and invited to one of two online meetings in October 2020 during which they were introduced to the tool, the rationale for its development, and then asked for feedback. Initial feedback on usability and utility was provided by two smaller groups (*n* = 12 and 12) with novice implementers and implementation science researchers (i.e., less than 1 years’ experience or training in implementation science or implementation) and one experienced group (i.e., more than 1 years’ experience or training) (*n* = 8). Participants in these meetings represented experience and expertise across multiple relevant areas. As per recommendations [16], the proportion of content experts was greater than 25%.

Meetings began with a presentation on relevant background topics, including a summary of the evidence on existing tools and a summary of the progress in consensus building among the expert panel to develop the current items presented in the tool. Meetings were moderated by one expert panel member while 1–2 team members took notes. The discussions were recorded. Analysis of discussion notes was conducted by NR, and findings were shared with the expert panel for interpretation. In addition to verbal feedback, participants were invited to complete questionnaires (*n* = 9). Data management and analysis was carried out in Excel 2016. An audit trail was generated to capture the progression of the tool development and decisions that were made regarding additions or edits to its components and structure. At the end of each meeting, the expert panel sought feedback on a knowledge translation strategy.

### Phase 3: Post-meeting activities

After the meetings, the expert panel reconvened with an online meeting to debrief on the larger consensus generating meetings, including voting on suggested modifications for usability and automation. The panel began work on implementing a knowledge translation strategy, including preparing publication of the tool and an explanation and elaboration document and development of a website to host the tool (https://publichealth.nyu.edu/research-scholarship/centers-labs-initiatives/isee-laboratory).

## Results

The tool domains are identified below, including the description of the intervention and implementation strategy methods, the description of the intervention and implementation strategy results, the evaluation of intervention and implementation strategy methods, and the evaluation of the intervention and implementation strategy results. The instructions for its use are shared in Table 2. The 24 items are applicable to a broad range of study designs employed in implementation science, including qualitative studies, randomized-control trials, non-randomized quantitative studies, and mixed methods studies. A key feature of the tool is the dual columns for implementation strategy and intervention, within which the methods and results are described and the intervention and implementation outcomes are assessed for bias. Accompanying instructions and an elaboration document that identifies and describes each of the items, explains the rationale, and models examples of good reporting and appraising practice, as well as an instructional video were prepared.

**Table 2:** ASSESS tool item descriptions

#	Item	Description
1	Review or meta-analysis question	The overall question guiding the review or meta-analysis
	Notes	Use this space to write notes to yourself or other extractors regarding decisions on how to enter data
	Reported on page #	When used as a reporting tool, indicate the page number where the indicated information can be found
	ARTICLE CITATION	
2	Study author, publication year	Indicate the study author name and publication year
3	Study title	Indicate the study title
	INTRODUCTION	
	Implementation strategy	“Implementation strategy” refers to how the intervention was implemented.
	Intervention strategy	“Intervention” refers to the healthcare or public health intervention that is being implemented.
4	Rationale	For implementation strategy: the scientific background and rationale for the implementation strategy (including any underpinning theory/framework/model, how it is expected to achieve its effects and any pilot work). For intervention: the scientific background and rationale for the intervention being implemented (including evidence about its effectiveness and how it is expected to achieve its effects).
5	Aim(s), objective(s), or research question(s)	Are there clear aims, objectives, or research questions? Indicate the primary (upon which the study was primarily designed to address) and the secondary (addressing this is prioritized after the primary)
	METHODS: DESCRIPTION	
6	Descriptions	A description of the intervention and implementation strategy. Identify the components that are core components vs those that are tangential and modifiable for the context, if possible.
7	Adaptation	A description of any adaptation that has and/or will occur.
8	Design	The design and key features of the evaluation, (cross referencing to any appropriate methodology reporting standards) and any changes to study protocol, with reasons
9	Participant types	Who are the participants in the intervention and implementation strategy
10	Comparison group	If experimental design, indicate the comparison group for the intervention and/or implementation strategy
11	Context	The context in which the intervention was implemented.
12	Sites	The characteristics of the targeted ‘site(s)’ (e.g locations/personnel/resources etc.) for implementation and any eligibility criteria.
13	Subgroups (optional)	Any sub-groups recruited for additional research tasks, and/or nested studies are described
14	Implementation phase	Indicate whether evaluation occurred pre-, during , and/or post-implementation
15	Process evaluation	Process evaluation objectives and outcomes related to the mechanism by which the strategy is expected to work
16	Sample size	Rationale for sample sizes (including sample size calculations, budgetary constraints, practical considerations, data saturation, as appropriate)
17	Analysis	Methods of analysis (with reasons for that choice)
18	Sub-group analyses	Any a priori sub-group analyses (e.g. between different sites in a multicenter study, different clinical or demographic populations), and sub-groups recruited to specific nested research tasks
19	Outcomes (assessment) (Implementation)	Defined pre-specified primary and other outcome(s) of the implementation strategy, and how they were assessed. Document any pre-determined targets
	Quantitative column	Input the specific outcome
	Qualitative column	Input the specific outcome
	Acceptability	the perception among implementation stakeholders (beneficiaries and implementers) that the innovation is agreeable, palatable, or satisfactory
	Adoption	the intention, initial decision, or action to try or employ the innovation (i.e. uptake)
	Appropriateness	the perceived fit, relevance, or compatibility of the innovation for a given practice setting, provider, or beneficiary; and/or perceived fit of the innovation to address a particular issue or problem (OH prevention).
	Feasibility	the extent to which the innovation can be successfully used or carried out within a given agency or setting
	Fidelity	degree to which the innovation can be implemented as it was prescribed in the original protocol or as it was intended by the program developer
	Cost	(incremental or implementation cost) is defined as the cost impact of an implementation effort
	Penetration	the integration of a practice within a service setting and its subsystems
	Sustainability	the extent to which a newly implemented innovation is maintained or institutionalized within a service setting’s ongoing, stable operations
19	Outcomes (assessment) (Intervention)	Defined pre-specified primary and other outcome(s) of the intervention (if assessed), and how they were assessed. Document any pre-determined targets
	Quantitative column	Input the specific outcome
	Qualitative column	Input the specific outcome
	Effectiveness	provision of services based on scientific knowledge to all who could benefit and refraining from providing services to those not likely to benefit (avoiding underuse and overuse, respectively)
	Efficiency	the avoidance of waste, including waste of equipment, supplies, ideas,and energy
	Equity	provision of care that does not vary in quality because of personalcharacteristics such as gender, ethnicity, geographic location, and socioeconomic status
	Patient centeredness	provision of care that is respectful of and responsive to individual patient preferences, needs, and values and ensuring that patient values guide all clinical decisions
	Safety	the avoidance of injuries to patients from the care that is intended to help them
	Timeliness	reduction of waits and sometimes harmful delays for both thosewho receive and those who give care
	RESULTS: DESCRIPTION	
20	Outcomes (findings) (Implementation)	Defined pre-specified primary and other outcome(s) of the implementation strategy, and how they were assessed. Document any pre-determined targets
	Quantitative column	Input the specific outcome
	Qualitative column	Input the specific outcome
	Acceptability	the perception among implementation stakeholders (beneficiaries and implementers) that the innovation is agreeable, palatable, or satisfactory
	Adoption	the intention, initial decision, or action to try or employ the innovation (i.e. uptake)
	Appropriateness	the perceived fit, relevance, or compatibility of the innovation for a given practice setting, provider, or beneficiary; and/or perceived fit of the innovation to address a particular issue or problem (OH prevention).
	Feasibility	the extent to which the innovation can be successfully used or carried out within a given agency or setting
	Fidelity	degree to which the innovation can be implemented as it was prescribed in the original protocol or as it was intended by the program developer
	Cost	(incremental or implementation cost) is defined as the cost impact of an implementation effort
	Penetration	the integration of a practice within a service setting and its subsystems
	Sustainability	the extent to which a newly implemented innovation is maintained or institutionalized within a service setting’s ongoing, stable operations
20	Outcomes (findings) (Intervention)	Defined pre-specified primary and other outcome(s) of the intervention (if assessed), and how they were assessed. Document any pre-determined targets
	Quantitative column	Input the specific outcome
	Qualitative column	Input the specific outcome
	Effectiveness	provision of services based on scientific knowledge to all who could benefit and refraining from providing services to those not likely to benefit (avoiding underuse and overuse, respectively)
	Efficiency	the avoidance of waste, including waste of equipment, supplies, ideas,and energy
	Equity	provision of care that does not vary in quality because of personalcharacteristics such as gender, ethnicity, geographic location, and socioeconomic status
	Patient centeredness	provision of care that is respectful of and responsive to individual patient preferences, needs, and values and ensuring that patient values guide all clinical decisions
	Safety	the avoidance of injuries to patients from the care that is intended to help them
	Timeliness	reduction of waits and sometimes harmful delays for both thosewho receive and those who give care
21	Barriers to implementation	Identify any factors examined that do or could challenge successful implementation
22	Facilitators of implementation	Identify any factors examined that do or could support successful implementation
	METHODS: EVALUATION	
23	Design	Follow steps 1, 2, and 3
	Step 1	Insert design type: Qualitative, Quantitative RCT, Quantitative non-randomized, or Mixed methods
	Step 2	Step 2 insert 5 corresponding criteria from instructions
	Qualitative criteria	1.1. Is the qualitative approach appropriate to answer the research question?
		1.2. Are the qualitative data collection methods adequate to address the research question?
		1.3. Are the findings adequately derived from the data?
		1.4. Is the interpretation of results sufficiently substantiated by data?
		1.5. Is there coherence between qualitative data sources, collection, analysis and interpretation?
	Quantitative, RCT criteria	2.1. Is randomization appropriately performed?
		2.2. Are the groups comparable at baseline?
		2.3. Are there complete outcome data?
		2.4. Are outcome assessors blinded to the intervention provided?
		2.5 Did the participants adhere to the assigned intervention?
	Quantitative, non-randomized criteria	3.1. Are the participants representative of the target population?
		3.2. Are measurements appropriate regarding both the outcome and intervention (or exposure)?
		3.3. Are there complete outcome data?
		3.4. Are the confounders accounted for in the design and analysis?
		3.5. During the study period, is the intervention administered (or exposure occurred) as intended?
	Mixed methods criteria	4.1. Is there an adequate rationale for using a mixed methods design to address the research question?
		4.2. Are the different components of the study effectively integrated to answer the research question?
		4.3. Are the outputs of the integration of qualitative and quantitative components adequately interpreted?
		4.4. Are divergences and inconsistencies between quantitative and qualitative results adequately addressed?
		4.5. Do the different components of the study adhere to the quality criteria of each tradition of the methods involved?
	Step 3	Provide score (0 or 1) to each criteria where 1 indicates that the criteria was met and 0 indicates the criteria was not met
	RESULTS: EVALUATION	
	Step 4	Sum the score from Step 3 and apply to the outcomes assessed
24	Outcomes	
	Bias Column	Indicate the degree of bias based on the design and methods, where 1-2=higher bias and 3-5=lower bias. Can also input 'unclear' if the degree of bias cannot be determined, or 'NA' for outcomes not assessed

### Intervention and implementation description: methods

This is the first domain (items 1–19), which tasks the user with describing the implementation strategy and intervention implemented, including the following items: (1) overall review or meta-analysis question, (2) study author and publication year, (3) study title, (4) rationale, (5) aim(s), objective(s), or research question(s), (6) description of the intervention and/or implementation strategy, (7) description of any adaptation of the intervention or its delivery, (8) study design, (9) participant type(s), (10) comparison group, (11) context, (12) study sites, (13) subgroups (optional), (14) implementation phase, (15) process evaluation, (16) sample size, (17) analysis, (18) sub-group analyses (optional), and (19) outcomes (assessment). The user enters how data was collected for assessment of both implementation outcomes (i.e., acceptability, appropriateness, adoption, feasibility, fidelity, penetration, cost, and sustainability) and intervention outcomes (i.e., effectiveness, efficiency, equity, patient-centeredness, safety, and timeliness), recommended by Proctor et al. [8] as relevant.

### Intervention and implementation description: results

The next domain (items 20–22) is where the user describes the results of both the implementation strategy and intervention implemented. As appropriate, the user enters [20] outcomes (implementation and intervention outcomes), [21] barriers to implementation, and [22] facilitators of implementation.

### Intervention and implementation evaluation: methods

The third domain (item 23) is where the user evaluates the methods reported within the paper to assess the implementation strategy and the intervention implemented. The tool guides the user through this process in three steps. First, the user selects the study design (i.e., qualitative, randomized control trial, non-randomized quantitative study, or mixed methods). Next, the user is prompted to respond to five questions regarding the study design reported in the paper. Criteria are indicated by study design, so that criteria for qualitative studies correspond to criteria 1.1–1.5, those for quantitative RCTs correspond to 2.1–2.5, those for quantitative non-randomized studies correspond to 3.1–3.5, and those for mixed methods studies correspond to 4.1–4.5. Each question represents a quality criterion for evaluating the study design. For qualitative studies, for example, the criteria are as follows: 1.1. Is the qualitative approach appropriate to answer the research question?; 1.2. Are the qualitative data collection methods adequate to address the research question?; 1.3. Are the findings adequately derived from the data?; 1.4. Is the interpretation of results sufficiently substantiated by data?; 1.5. Is there coherence between qualitative data sources, collection, analysis and interpretation? In comparison, for the quantitative RCTs, the criteria are as follows: 2.1. Is randomization appropriately performed?; 2.2. Are the groups comparable at baseline?; 2.3. Are there complete outcome data?; 2.4. Are outcome assessors blinded to the intervention provided?; 2.5 Did the participants adhere to the assigned intervention? Finally, the user provides a score (0 or 1) to each question, to indicate whether each criterion was (1) or was not (0) met.

### Intervention and implementation evaluation: results

The last domain (item 24) is where the user inputs their evaluation of the results of the implementation strategy and the intervention implemented. The user sums the score from the last step of the third domain and applies this summary score to the intervention and implementation outcomes assessed. Based on this appraisal section, the risk of bias will be higher (i.e., score of 1–2), lower (i.e., score of 3–5), or unclear (i.e., not able to be assessed). A summary score can be applied to each implementation or intervention outcome assessed in the paper, if these outcomes were assessed in different manners. For example, a study may have poorly evaluated the intervention outcome (i.e., summary score for effectiveness = 2 and for patient centeredness = 1) but appropriately evaluated the implementation outcome (i.e., summary score for adoption = 4 and for acceptability = 5). Additionally, summary scores may be compared across various studies within a review, which will provide an overall understanding of the risk of bias within the literature for each outcome of an intervention and implementation strategy. This synthesis and appraisal can be guided by the templates included as supplementary documents. As with standards for systematic reviews, it is advised that at least two reviewers independently carry out the appraisal process and compare extraction until reaching consensus or have a third reviewer resolve discordant outputs.

### Usability and utility findings

Once the tool was developed, feedback was sought on its usability and utility. Our sample of 32 meeting participants were majority female, with a mix of education attainment, across various healthcare and public health disciplines, and ranged from novice to expert implementers and researchers. (Table 3) Users reported they liked the layout of the tool, its detailed instructions, and ease of use (Table 4). Many reported it was comprehensive and saw utility in being able to extract both qualitative and quantitative results, with one participant sharing “I am very excited about this tool because I am working on a literature review and have been having trouble thinking about how to organize the evidence to inform implementation science.” Participants also recognized that this made for a lengthy extraction process. Another participant shared: “I think this is really useful. It would be great to employ this in several different disciplines to see how it works in real practice.” They found the criteria scoring for critical appraisal were straightforward. Participants asked for examples of completed entries and wanted to have space to identify the individual entering information in the form, so one could assess inter-rater reliability. Many had suggestions for how to improve automation.

**Table 3:** Sample characteristics of consensus meeting participants regarding usability testing (*N* = 32)

Characteristic	*N* (%)
Gender	27 (84%) female
Education	11 (34%) bachelors
	11 (34%) masters
	10 (31%) MD or PhD
Disciplines	18 (56%) students
	10 (31%) researchers
	2 (6%) physicians
	1 (3%) nurse
	1 (3%) postdoc
	1 (3%) professor
	1 (3%) hospital administrator
	1 (3%) nutritionist
	1 (3%) allied health professional
	1 (3%) clinical manager
Implementation science experience	3 (9%) formal implementation science training
	7 (32%) among researchers, those who use Implementation Science frameworks/theories
	5 (63%) among those engaged in Implementation Science research with 1 year or less of experience

**Table 4:** Participant feedback on utility and usability (*N* = 9)

Statement	% agree/strongly agree
This tool is **helpful for designing and carrying out systematic reviews or meta-analyses** of empirical studies of implementation.	100%
This tool **addresses the challenge of critical assessment of implementation outcomes** within a methodologically diverse and growing body of implementation science literature.	100%
This tool could **support examining how process and context may lead to heterogeneity of results** in implementation research.	89%
The use of this tool could **improve the reporting and synthesis of implementation strategies**.	100%
I am **likely to use this tool** once it is available.	78%
As this tool **seems too burdensome**, I would be unlikely to use it.	33%

### Automation

The team is in the process of investigating existing platforms that will facilitate automation. It is noted that reviewers will have access to different tools, thus Excel will be the primary tool. This will allow the tool to interface with statistical packages to enable the generation of summary statistics when comparing across multiple extracted papers within a systematic review or meta-analysis. Editing capabilities specific to the process will be incorporated into future tools.

## Discussion

The comprehensive, adaptable 24-item ASSESS tool allows for both (1) reporting of implementation strategies and the intervention being implemented and (2) critical appraisal of intervention and implementation outcomes resulting from quantitative, qualitative, or mixed methods assessment. The tool shares with the STARI checklist [20] the aim for enhancing adoption and sustainability of effective interventions by structuring reporting of implementation studies, as well as the presentation of dual strands describing the implementation strategy and the intervention that is being implemented. The ASSESS tool is novel in its inclusion of the implementation phases, which allows for comparison of studies across pre-implementation, during implementation, and post-implementation stages. These stages could then be mapped onto implementation science theoretical frameworks like the Exploration, Preparation, Implementation and Sustainment (EPIS) Framework, to generate findings related to applicability of implementation strategies and assessment of implementation outcomes at different implementation phases. The ASSESS tool is innovative in that other reporting tools generally do not assess risk of bias among implementation outcomes. Other critical appraisal tools do not provide guidance on how to separately assess quantitative and qualitative data for risk of bias. Shaped by Proctor’s taxonomy, the ASSESS tool moves from simply reporting implementation outcomes to evaluating quality of data on the outcomes and thus the risk of bias. The ASSESS tool will need to be refined in the light of the practical experience of using the tool. Further research is needed to examine how to integrate quantitative (risk of bias) and qualitative (trustworthiness) if critical appraisal findings are discordant.

This tool has various strengths and limitations. As a strength, it does not promote one study design over another, for example randomized-control trial over qualitative. It provides a way to appraise qualitative findings, which are somewhat lesser reported than quantitative appraisal. It further incorporates implementation phases. Importantly, this work presents the development of the tool and initial qualitative assessment; it is only once it is available for use that its greater utility can be subsequently assessed. Future research should examine the validity and reliability of the ASSESS tool, as has been done using a stakeholder-driven approach for pragmatic measurement of implementation outcomes, strategies, and context [11, 12, 24]. Future research should also examine tool iterations that integrate aspects of additional novel and relevant tools, such as the FRAME-IS tool for documenting modifications to implementation strategies in healthcare [25], which was published after our work was carried out and therefore did not inform the consensus building process. This tool is not designed for use with non-empirical papers (i.e., review papers, theoretical papers, gray literature where the methods are not fully described), economic studies, or diagnostic accuracy studies. Future research may examine iterations of this tool to allow application to these types of studies, as well as examine the variability of qualitative designs for critical appraisal. Although a Delphi method may provide more reliable findings, there are certain advantages to using nominal groups, including greater consensus and understanding of reasons for disagreement; therefore, elements of a modified Delphi method and a nominal group technique were combined through a hybrid method that has been previously suggested [26]. These structured methods attempt to combat cognitive biases in judgment [27], which are particularly influential in complex tasks [19], as both require an independent initial rating to anchor opinions based on an individual’s own knowledge. This hybrid method maintained a focused discussion on specific topics pertinent to the underlying validity of each item in the tool and allowed all panelists to have access to the same information regarding the tool prior to evaluating it.

## Conclusion

The comprehensive, adaptable 24-item ASSESS tool allows for both (1) reporting of the implementation strategy and the intervention being implemented and (2) critical appraisal of intervention and implementation outcomes resulting from quantitative, qualitative, or mixed methods assessment. It addresses the challenge of critical assessment of a methodologically diverse and growing body of implementation science literature. This tool could prove particularly helpful for designing and carrying out systematic reviews and meta-analyses of empirical studies of implementation, examining how process and context may lead to heterogeneity of results. The ASSESS tool will be disseminated via posting on the researchers’ website (https://publichealth.nyu.edu/research-scholarship/centers-labs-initiatives/isee-laboratory) and via submission to the Enhancing the QUAlity and Transparency Of health Research (EQUATOR) Network. Its use could improve the synthesis of implementation strategies, which will facilitate translation of effective public health interventions into routine practice within clinical or community settings.

## Data Availability

The tool will be available on a website (https://publichealth.nyu.edu/research-scholarship/centers-labs-initiatives/isee-laboratory). Templates in various forms will be made available.

## Abbreviations

ASSESS: A comprehenSive tool to Support rEporting and critical appraiSal of qualitative quantitative and mixed methods implementation reSearch outcomes
